# The Pursuit of Approval: Social Media Users’ Decreased Posting Latency Following Online Exclusion as a Form of Acknowledgment-Seeking Behavior

**DOI:** 10.1177/01461672241297824

**Published:** 2024-11-22

**Authors:** Christoph Kenntemich, Christiane M. Büttner, Selma C. Rudert

**Affiliations:** 1 University of Kaiserslautern-Landau, Germany; 2 University of Basel, Switzerland

**Keywords:** social exclusion, ostracism, social media, behavioral coping

## Abstract

How do individuals behave after the sting of social exclusion on social media? Previous theorizing predicts that, after experiencing exclusion, individuals either engage in activities that reconnect them with others, or, they withdraw from the context. We analyzed data from Twitter (*k* = 47,399 posts; *N* = 2,000 users) and Reddit (*k* = 58,442 posts; *N* = 2,000 users), using relative (un)popularity of users’ own posts (i.e., receiving fewer Likes/upvotes than usual) as an indicator of social exclusion. Both studies found no general increase or decrease in posting latency following exclusion. However, the latency of behaviors aimed at connecting with *many others* decreased (i.e., posting again quickly), and the latency of behaviors aimed at connecting with *specific others* increased (i.e., commenting or mentioning others less quickly). Our findings speak in favor of acknowledgment-seeking behavior as a reaction to social exclusion that may be specific to social media contexts.

Social media has become an integral part of human life, including the way that social interactions are lived (e.g., Büttner et al., 2023; Reich et al., 2018). Social media offers novel ways of connecting with other people, simplifying social interactions into paralinguistic digital affordances such as Likes, hearts, “+1,” and emojis (e.g., Hayes et al., 2016). However, these novel forms of social interaction also pave the way for negative experiences, such as novel forms of exclusion (i.e., feeling ignored and excluded by others on social media, e.g., Williams, 2009; Wolf et al., 2015). For instance, not getting a satisfactory number of paralinguistic digital affordances such as Likes in response to a social media post is perceived as social exclusion (e.g., Lutz & Schneider, 2020; Reich et al., 2018), even in fictional social media networks (e.g., Wolf et al., 2015). Following these observations, we use the relative (un)popularity of users’ own posts (i.e., receiving fewer Likes/upvotes than usual) as an indicator of social exclusion. Social exclusion has highly aversive consequences for its targets both in offline and online contexts, such as threatening needs of belonging, control, self-esteem, and meaningful existence (e.g., Büttner & Rudert, 2022; Williams, 2009; Wolf et al., 2015). If not remedied, social exclusion fosters depression and suicidality in the long run (e.g., Büttner & Greifeneder, 2024; Chen et al., 2020). Importantly, social exclusion is distinct from experiencing negative attention, such as rejection or bullying: Social exclusion is the absence of attention and is more emotionally aversive than rejection (Rudert et al., 2017) or bullying (Zadro et al., 2005). Social exclusion represents being rendered meaningless, a severely threatening experience compared with receiving negative attention where at least the target receives some attention (e.g., Rudert et al., 2017; Williams & Nida, 2009). On social media, not receiving a usual amount of Likes, an exclusionary experience, is equally threatening to needs for meaningful existence and control as receiving Dislikes, that is, negative attention (but receiving Dislikes threatened needs for belonging and self-esteem more strongly than social exclusion, Lutz & Schneider, 2020).

To cope with social exclusion and recover their threatened needs, individuals can engage in various behaviors, such as seeking reconnection with others, aggressing against them, or withdrawing from social interactions altogether (e.g., Maner et al., 2007; Ren et al., 2018, 2020). Social media also offers features that may help excluded individuals cope with their experience, such as posting content or leaving Likes, Dislikes, or comments under other users’ posts (e.g., Karahanna et al., 2018; Lutz et al., 2022, 2023). A shared problem of previous research into behavioral consequences of social exclusion, in the real world and in social media contexts, is that the often-used laboratory studies come at a cost of decreased external validity of participants’ assessed behaviors (see, for example, Baumeister et al., 2007). We propose to close this gap with observational data from two social media platforms: Twitter (at the time of data mining, now “X”) and Reddit. Twitter is a micro-blogging social media platform where users post *tweets* (short texts) and interact with other users through *favorites* (liking another user’s post), *retweets* (reposting another user’s post), *mentions* (addressing another user by linking their profile name in direct response or in a comment), and *comments* that respond to another user’s post (e.g., Karahanna et al., 2018). Reddit is a social media platform focused on content sharing and discussion among users. Reddit users may post links, videos, news, pictures, or texts in *subreddits* (forums ordered by topic) where other users can respond via *comments* and interact with *upvotes* or *downvotes* to the post, indicating popularity of the content (e.g., Proferes et al., 2021).

We formulate three hypotheses on how individuals may behave after being socially excluded on social media: Users may behave to reconnect with *many* others and behave to reconnect with *specific individuals*, or they may withdraw from the situation. We review arguments and empirical evidence for each hypothesis in the following.

**Hypothesis 1: The “Reconnection with Many” Hypothesis**. Social exclusion disconnects individuals from others, causing them to seek reconnection (e.g., Eck et al., 2016; Williams, 2009). Social media is a unique coping tool in that it offers the possibility to connect with *many* individuals simultaneously, for instance by posting content for everyone to see (e.g., Karahanna et al., 2018). By posting content, social media users may express themselves and exert control over how they want others to perceive them (e.g., as highly knowledgeable, popular, or connected, see, for example, Büttner et al., 2023). Posting content may also be an effective tool in restoring relatedness and other needs threatened by social exclusion (e.g., Lutz et al., 2022). We conceptualize posting to reconnect with *many* others as behaving to be *acknowledged* rather than to be *liked* after exclusion on social media. Relating to Williams’s (2009) Temporal Need Threat Model, exclusion on social media, a context where individuals seek acknowledgment from others (Karahanna et al., 2018), threatens power and provocation needs (i.e., needs for control and for being acknowledged in one’s existence by others, Williams, 2009) more than inclusionary needs (i.e., needs for belonging and self-esteem, Williams, 2009), in line with previous experimental findings (Lutz & Schneider, 2020). Thus, with a mass social media audience, the quality of exclusion encapsulated by few Likes could lead to a specific deficit in acknowledgment that is qualitatively different from other exclusions. To re-afford these needs, actions that are aimed at being acknowledged by many others might be more effective for individuals’ coping than actions aimed at reconnecting with specific others. In summary, users who just experienced exclusion on social media may post content faster to a large audience to increase their chances of reconnecting with many others.
**Hypothesis 2: The “Reconnection with Specific Individuals” Hypothesis.** Instead of reconnecting with a vast number of individuals, the Reconnection with Specific Individuals Hypothesis holds that excluded individuals may turn to specific persons for reconnection. Specifically addressing another user in a comment, or mentioning them, might be more effective to prompt an answer from that person and thus connect with them, than posting content and hoping for one out of an unspecified throng of people to respond. On Twitter, a mention is a post that is either made in direct response to another user’s post (i.e., @user followed by a comment under that user’s post) or that otherwise addresses another user directly (i.e., @user in a post). A retweet on Twitter echoes another user’s post to the reposting user’s followers. Both mentions and retweets may evoke reciprocal communication: Reciprocity is a strong interaction norm that holds that individuals employ a “mutually contingent exchange of gratifications” (Gouldner, 1960, p. 161). Consistent with this notion, Twitter users retweet others’ content out of *reciprocity motivation*, that is, because they expect a message in return, or because their content had been previously retweeted (M. Lee, Kim, & Kim, 2015). Thus, the likelihood of receiving an answer from the user that one has mentioned or retweeted may be higher because of reciprocity norms than the likelihood that others respond to a post that is not tailored to one specific individual. Importantly, even being included by one single person effectively alleviates the negative impact of social exclusion (e.g., DeWall et al., 2010). Perceived chances of re-inclusion also shape excluded individuals’ behavioral reactions (Cuadrado et al., 2015). Chances of re-inclusion may be higher when approaching specific others versus a large group of social media users, which may cause excluded individuals to choose the former strategy over the latter. Moreover, commenting on other users’ posts or mentioning other users and their content may already boost one’s relatedness needs (i.e., meta-voicing, Karahanna et al., 2018). In conclusion, users who experienced exclusion may engage in reconnection behavior aimed at *specific individuals* such as commenting or sharing other users’ content.
**Hypothesis 3: The Withdrawal Hypothesis.** Experimental research documents large effects of social exclusion on withdrawal from others (e.g., Ren et al., 2020). These effects may seem contradictory given the evidence reviewed above on reconnection efforts after exclusion. However, by withdrawing, socially excluded individuals may protect themselves against future negative experiences in the same environment (e.g., Ren et al., 2020). Social media is a context that is especially easy to leave without immediate negative consequences (as opposed to, leaving a work meeting, or a school lesson). Therefore, it could also be that, following social media exclusion, individuals withdraw and refrain from using social media for some time to seek connections elsewhere.

## The Present Research

Understanding how users respond to unfavorable reception in social media environments is a pressing research question. Using data from Twitter and Reddit, we test whether the users’ time until the next post or comment increased or decreased following exclusion, in line with the idea that posting and commenting represent immediate coping responses to exclusion.

### Operationalizations

We analyzed users’ online behaviors following social exclusion, defined by a lack of popularity of users’ posts compared with the usual popularity of a user’s post (see Lutz & Schneider, 2020; Reich et al., 2018). Particularly, we focused on the latency until users’ next post or comment after an unpopular post. We operationalized shorter latency (less time) until a user’s next post as indicative of attempts to *reconnect with many* other social media users, shorter latency until a user’s next mention, retweet (Study 1, Twitter), or comment (Study 2, Reddit) as indicative of attempts to *reconnect with specific individuals*, and, finally, we operationalized longer latency (more time) until a user’s next post as indicative of *withdrawal* from social media.

### Unpopular Versus Regularly Popular Posts

Importantly, we hypothesize that the effects are primarily influenced by social exclusion. If social exclusion is the driving factor, the behavioral differences should be more pronounced when contrasting an unusually unpopular post with a regularly popular one, as opposed to comparing an exceptionally popular post with one of average popularity. That is, because we expect the effects to be driven by social exclusion, all of the predicted relations are not necessarily linear; we only predict differences between a particularly low number of paralinguistic digital affordances (i.e., in the lower third of the distribution, what we define as a point of minimal social disappointment) versus a not-low number of paralinguistic digital affordances (i.e., the two upper thirds of the distribution). We contrast the lowest third with the two upper thirds because unexpectedly popular posts (i.e., the upper third) are not the focus of this contribution. The choice of the lower third of the distribution as the point of minimal social disappointment was data-driven since the tertile split fit the data better than a median split, quartile split, or a cubic predictor (see Open Science Framework [OSF] Supplement 1a—l. 419ff). However, a continuous quadratic predictor was also a good fit compared with a baseline model (see OSF Supplementary model fits). Therefore, we explore quadratic relationships in exploratory fashion in both studies.

### Method Statement

#### Open Science Practices

We report all data exclusions, data manipulations, and measures. All code for data mining, data preparation, and statistical analyses, as well as the data for both studies is available via the OSF (https://osf.io/h62gz/?view_only=13360594eb654ab4a690ebdd11a4da00). We preregistered all hypotheses, sample sizes, exclusion criteria, and analysis plans on AsPredicted (Study 1: https://aspredicted.org/kt8v-jrhx.pdf; Study 2: https://aspredicted.org/ndvn-276b.pdf).

#### Data Mining

As an indicator of social exclusion, we focused on the *relative* popularity of users’ *own content*. Many experimental exclusion paradigms rely on social comparison with other individuals (e.g., receiving fewer Likes than other social media users, Wolf et al., 2015). Here, the users’ own previous posting experiences serve as the reference point, comparing users’ post popularity to their own previous posts. To create our predictor variable, we excluded all posts in which users mentioned others, or retweeted other people’s content on Twitter (Study 1), and we only analyzed posts as a predictor (as opposed to comments on others’ posts) on Reddit (Study 2). To allow for intraindividual comparisons, we excluded individuals with less than 10 posts in total. To ensure the normality of our data and to focus on the range of theoretical interest, as preregistered, we excluded uncommonly popular posts from our sample. To do so, first, we transformed the independent variable (i.e., in Study 1: X = 2 × √favorite count, in Study 2: X = 2 × √score). Next, following a median absolute derivation approach (e.g., Howell, 2005), we defined uncommonly popular posts as exceeding a set robust threshold for count data, that is, X > Median(X) + 10. While a more typical threshold for extreme value identification is set at 3 units above the median, that is, X > Median(X) + 3, we adopted a more liberal criterion to account for potential low gamma of the distribution and exclude extremely popular posts exclusively. This excluded roughly the most popular 13% of posts in the Twitter sample and the most popular 30% in the Reddit sample.^1^ This exclusion criterion was not preregistered for Study 1 but was preregistered for Study 2. In exploratory fashion, we explore the analyses again in the full data set, without excluding extremely popular posts (Tables 1 and 4).

**Table 1.** Results of the LMEMs in Study 1, With Filtered (i.e., Excluding Extremely Popular Posts) and Unfiltered (i.e., Full Data Set) Data.

Data	Latency variable	Main effect *b* [95% CI]	Main effect—% (+1 *SD*/−1 *SD*)	Lower third *b* [95% CI]	Lower third—% (+/−1 *SD*)	Upper two thirds *b* [95% CI]	Upper two thirds—% (+/−1 *SD*)	Quadratic term *b* [95% CI]	Fixed effects (*SE*)	Random effects (*SE*)
Filtered	Next post	**0.09 [0.07, 0.11]**, ***p* < .001**	**+09.64%/**−**8.79%**	**0.16 [0.12, 0.20]**, ***p* < .001**	**+17.22%/**−**14.69%**	**0.05 [0.02, 0.07]**, ***p* = .001**	**+4.77%/**−**4.56%**	−**0.03 [**−**0.04**, −**0.01]**, ***p* < .001**	***β_0_* = 11.93 (0.04)**, ***p*** **< .001**; ***β1* = 0.05 (0.01)**, ***p*** **= .001**; ***β2* = 0.11 (0.03)**, ***p*< .001**	*u_0j_* = 2.71; *u_1j_* = 0.03; Cov(*u_0j_, u_1j_*) = −0.26; *r_ij_* = 3.47
	Next mention	−**0.15 [**−**0.17**, −**0.12]**, ***p <* .001**^†^	−**13.50%/+15.60%**	−0.06 [−0.11, 0.02], *p* = .054	−6.17%/+6.58%	−**0.20 [**−**0.23**, −**0.16]**, *p* **< .001**	−**18.03%/+22.00%**	−**0.03 [**−**0.05**, −**0.02]**, *p* **< .001**^†^	***β_0_* = 11.03 (0.05)**, ***p*** **< .001**; ***β1* =** −**0.20 (0.02)**, ***p*** **< .001**; ***β2* = 0.14 (0.03)**, ***p*** **< .001**	*u_0j_* = 3.60; *u_1j_* = 0.06; Cov(*u_0j_, u_1j_*) = −0.01; *r_ij_* = 5.11
	Next retweet	**0.04 [0 .02, 0.06]**, ***p* = < .001**	**+3.67%/**−**3.54%**	**0.08 [0.04, 0.12]**, ***p* < .001**	**+8.44%/**−**7.78%**	0.01 [−0.02, 0.04], *p* = .666	+0.63%/−0.63%	−**0.01 [**−**0.03**, −**0.00]**, ***p* = .049**	***β_0_* = 11.94 (0.04)**, *p* **< .001**; *β1* = 0.06 (0.01), *p* = .666; ***β2* = 0.07 (0.03)**, *p* **= .005**	*u_0j_* = 2.98; *u_1j_* = 0.02; Cov(*u_0j_, u_1j_*) = −0.15; *r_ij_* = 3.43
Unfiltered	Next post	**0.08 [0.06, 0.10]**, ***p* *<* .001**	**+8.55%/**−**7.87%**	**0.16 [0.11, 0.21]**, ***p* < .001**	**+17.20%/**−**14.67%**	**0.05 [0.03, 0.08]**, *p* **< .001**	**+5.49%/**−**5.21%**	−**0.03 [**−**0.04**, −**0.02]**, *p* **< .001**^†^	***β_0_* = 11.80 (0.04)**, ***p*** **< .001**; ***β1* = 0.05 (0.01)**, ***p*** **< .001**; ***β2* = 0.11 (0.03)**, ***p*** **< .001**	*u_0j_* = 1.56; *u_1j_* = 0.002; Cov(*u_0j_, u_1j_*) = −0.96; *r_ij_* = 4.63
	Next mention	−**0.20 [**−**0.22**, −**0.18]**, ***p* < .001**^†^	−**18.13%/+22.14%**	−**0.18 [**−**0.23**, −**0.12]**, ***p* < .001**^†^	−**16.16%/+19.27%**	−**0.21 [**−**0.24**, −**0.18]**, *p* **< .001**	−**18.67%/+22.95%**	0.00 [−0.01, 0.02], *p =* .484	***β_0_* = 10.95 (0.05)**, ***p*** **< .001**; ***β1* =** −**0.21 (0.02)**, ***p*** **< .001**; *β2* = 0.03 (0.04), *p =* .848	*u_0j_* = 1.82; *r_ij_* = 6.86
	Next retweet	0.02 [−0.002, 0.04], *p* = .072	+1.92%/−1.88%	**0.12 [0.07, 0.16]**, ***p* *<* .001**	**+12.39%/**−**11.03%**	−0.02 [−0.05, 0.005], *p* = .108	−2.10%/2.15%	−**0.02 [**−**0.03**, −**0.01]**, *p* ***<* .001**^†^	***β_0_* = 11.98 (0.04)**, ***p*** **< .001**; *β1* = −0.02 (0.01), *p* = .108; ***β2* = 0.14 (0.03)**, ***p*** **< .001**	*u_0j_* = 1.74; *r_ij_* = 4.71

*Note*. Significant effects are bold-faced. ^†^ Random-intercept only model. Parameters: intercept (*β_0_*), predictor favorite count (*β1*), discretizing factor (*β2*), intercept variance (*u_0j_*), slope variance (*u_1j_*), covariance (*u_0j_, u_1j_*), residual variance (*rij*).

#### Analytical Strategy

##### Mixed Effect Modeling

We conducted mixed effect modeling using the lme4 (Bates et al., 2015) and glmmTMB (Brooks et al., 2017) R-packages. We aimed for maximal linear mixed effect models (LMEMs), including random intercepts and random slopes, which are considered the gold standard (Barr et al., 2013). For exceptions when the maximal LMEM did not converge, we report the results from random-intercepts-only models. All reported *p* values are two-tailed.

The formula of the multilevel models is as follows:

$$\mathrm{Level}\;1\;\left(\mathrm{Within}\;\mathrm{Individuals}\right){:\;\mathrm{Latency}}_{ij}=\beta_{0j}+\beta_{1j}\left({\mathrm{Fav}}_{ij}\right)\;\;+\beta_{2j}\left({\mathrm{Fav}}_{ij}\times \;{\mathrm{Tertile}}_{ij}\right)\;+r_{ij}$$

Latency_*ij*_: Log-transformed latency to the next own post for post *i* of user *j*.

Fav_*ij*_: Z-score of the Anscombe-transformed favorites for post *i* of user *j*.

Tertile_*ij*_: Whether post *i* is in the lowest tertile of Favorites for user *j*.

*β_0j_*: Intercept for user *j.*

*β_1j_*: Slope of the effect of favorites on latency for user *j*.

*β_2j_*: Slope for interaction between favorites and being in the lowest tertile for user *j*.

*rij*: Residual for post *i* of user *j*.

$$\begin{array}{l} \mathrm{Level}\;2\;\left(\mathrm{Between}\;\mathrm{Individuals}\right):\beta_{0j}=\;\gamma_{00}+u_{0j};\\ \;\;\;\;\;\;\;\;\;\;\;\;\;\;\;\;\;\;\;\;\;\;\;\;\;\;\;\;\;\;\;\;\;\;\;\;\;\;\;\;\;\;\;\;\;\;\;\;\;\;\;\;\;\;\;\;\;\;\;\;\;\;\;\;\;\;\beta_{1j}=\;\gamma_{10}+u_{1j};\\ \;\;\;\;\;\;\;\;\;\;\;\;\;\;\;\;\;\;\;\;\;\;\;\;\;\;\;\;\;\;\;\;\;\;\;\;\;\;\;\;\;\;\;\;\;\;\;\;\;\;\;\;\;\;\;\;\;\;\;\;\;\;\;\;\;\;\beta_{2j}=\;\gamma_{20}+u_{2j} \end{array}$$

γ_00_: Overall intercept (latency when favorites and lowest tertile are both 0).

γ_10_: Slope of favorites’ effect on latency for user *j*.

γ_20_: Effect of being in the lowest tertile of favorites on the slope of favorites.

*u_0j_, u_1j_*, and *u_2j_*: Random effects capturing the deviation of user *j* from the average intercept (γ_00_), slope for Favorites (γ_10_), and the slope for the interaction term (γ_20_).

##### Variable Transformations

Latencies were log-transformed, that is, log (latency + 1) to improve linearity. Twitter favorites and Reddit scores were Anscombe-transformed to increase the normality of the highly skewed distribution (Anscombe, 1948) and standardized within individual (i.e., centering within cluster) to produce individual-level estimates (Enders & Tofighi, 2007). Degrees of freedom, test statistics, and *p* values were derived from Satterthwaite approximations in the lmerTest package (Kuznetsova et al., 2017). The exact formulas for all variable transformations can be found at the end of each of the R-Scripts (OSF).

##### Interpretation of Effect Sizes

We provide log-transformed regression coefficients and 95% confidence intervals (95% CI). Since all dependent variables were log-scaled, this coefficient can be interpreted as follows: For a post that is one standard deviation less popular, the decrease in time until the next post translates to a factor of e^−b^. So for *b* = 0.1: e^−0.1^ ≈ 0.905, corresponding to a 9.52% decrease. We report percentage decreases as an interpretation of the effect (Tables 1 and 4).

## Study 1

Study 1 explored responses to exclusion online with competing hypotheses on behavioral responses. We test the following hypotheses in Study 1:

**Hypothesis 1:** “Reconnection with Many”: The relative popularity of a tweet, operationalized as the number of favorites of a tweet relative to the usual number of favorites that a user receives, is positively associated with the time until posting the next tweet (posting latency).
**Hypothesis 2:** “Reconnection with Specific Individuals”: The relative popularity of a tweet is positively associated with (a) the time until the next mention (mentioning latency) and (b) the time until the next retweet (retweeting latency).

We preregistered that these relations are not necessarily linear, as we predict differences between a low number of favorites (exclusion) and a not-low count of favorites, and, therefore, no differences between an average amount of favorites and an above-average amount of favorites. In Study 1, we operationalized low number of favorites versus not-low number of favorites by exploratorily dividing the distribution into the lowest third versus the two upper thirds.

As a competing hypothesis to Hypotheses 1 and 2, we preregistered:

**Hypothesis 3:** “Withdrawal Hypothesis”: The relative popularity of a tweet is (a) negatively associated with time until posting the next tweet (posting latency), (b) time until the next mention (mentioning latency), and (c) time until the next retweet (retweeting latency).

### Method

#### Social Media Data

We sampled the latest 150 posts for a random sample of users and filtered them to maximize number of relevant data points per user and share of individual persons (as opposed to, organizations or companies; we excluded data from verified users^2^ and users that did not have a name as their profile name). We sampled roughly 10,000 users until 2,000 users met the criteria. For the full list of criteria, see OSF. The final data set consisted of 47,399 posts from 2,000 users and posted between August 2012 and November 2022. Data were randomly split into five random sets of 1,000 users to replicate the findings.

#### Measures

Please see OSF for the exact calculation of the different variables.

##### Favorite Count

The favorite count was Anscombe-transformed and standardized within individuals (i.e., centering within cluster) to produce individual-level estimates (Enders & Tofighi, 2007).

##### Latency Until Next Post

Latency until the next post indicates the time that passes until a user posts again, following their last post. For *threads* (i.e., a coherent string of posts), we only analyzed the first post to avoid misinterpretation of artificially low latencies until the next post in the thread.

##### Latency Until Next Mention

Latency until the next mention indicates the time that passes until a user posts a mention again, following their last post.

##### Latency Until Next Retweet

Latency until the next retweet indicates the time that passes until a user retweets a post, following their own last post.

### Results

#### Sample Characteristics

The median of users’ favorite count was 15.00 (*M* = 58.43), and the median user posted 1.39 times a week (*M* = 5.06). Information on users’ demographics (e.g., age, gender, and residence) is not available.

#### Latency Until the Next Post

We found a significant positive relationship between the popularity of the last post and the latency until the next post (Table 1). We introduced a discretizing factor, dividing the slope estimates into the lowest third of popularity (based on cluster-standardized measurements) and the upper two thirds representing more popular posts. We observe steeper positive slopes in the lowest third compared with the two upper thirds (Table 1).

**Figure 1.**
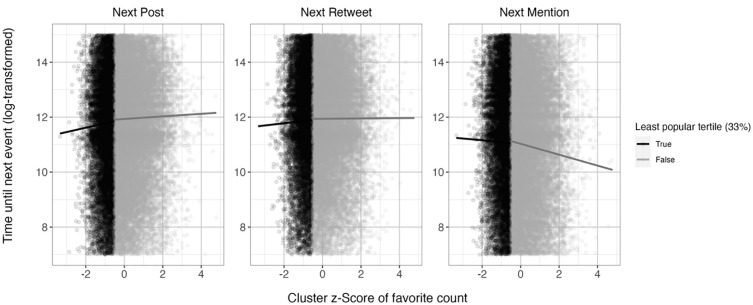
Association of Relative Favorite Count of a Post With the Time Taken Until the Next Own Tweet (Left Plot), Mention (Plot in the Middle), and Retweet (Right Plot), in Study 1. *Note.* Separate slopes are calculated for the least popular third of all posts versus the remaining posts.

Within-study replication confirmed a significant positive relationship between post popularity and latency until the next post, as well as steeper positive slopes in the lowest third compared with the two upper thirds (Table 2). Incorporation of this discretizing factor resulted in a significantly improved model fit, χ(1)^2^ = 18.95, *p* < .001. Our findings thus suggest a nonlinear relationship between the popularity of a post and the latency until the next post, with a steeper relationship in the lower third of the distribution (Figure 1). This suggests that users post more quickly following an unusually unpopular post, rather than posting particularly slowly following a very popular post. Findings thus align with the Reconnection with Many Hypothesis and suggest no evidence for the Withdrawal Hypothesis.

**Table 2.** Within-Study Replication Analyses for Studies 1 and 2.

Study	Variable	Share significant—main effect	Effect range —main effect	Share significant—lowest tertile	Effect range—lowest tertile	Share significant—upper two thirds	Effect range—upper two thirds	Share significant—quadratic	Effect range—quadratic
1	Latency next post	5/5 (100%)	0.07 to 0.10	5/5 (100%)	0.12 to 0.18	3/5 (60%)	0.02 to 0.05	4/5 (80%)	−0.04 to −0.02
	Latency next retweet^a^	5/5 (100%)	0.03 to 0.04	4/5 (80%)	0.04 to 0.09	0/5 (0%)	0.00 to 0.03	1/5 (80%)	−0.02 to −0.01
	Latency next mention	5/5 (100%)	−0.16 to −0.11	2/5 (40%)	−0.08 to −0.03	5/5 (100%)	−0.25 to −0.14	4/5 (80%)	−0.05 to −0.01
2	Latency next post (any subreddit)	5/5 (100%)	0.14 to 0.17	5/5 (100%)	0.24 to 0.35	4/5 (80%)	0.05 to 0.11	5/5 (100%)	−0.09 to −0.06
	Latency next post (same subreddit)	5/5 (100%)	0.14 to 0.15	5/5 (100%)	0.24 to 0.32	3/5 (60%)	0.05 to 0.08	5/5 (100%)	−0.09 to −0.07
	Latency next comment (other subreddit)	5/5 (100%)	−0.30 to −0.28	5/5 (100%)	−0.44 to −0.37	5/5 (100%)	−0.24 to −0.20	5/5 (100%)	0.04 to 0.06

*Note*. “Share Significant” describes how many out of the five within-study replication analyses yielded significant results. Reported effect ranges are regression coefficients (*b*). The effect in the lowest tertile tests whether the slope in that area is significantly different from zero (as opposed to significantly different from the upper two thirds). The “Main Effect,” is the slope of favorites (Twitter, Study 1) and score (Reddit, Study 2) *z* score in an LMEM without any further predictors, nonlinear or other. ^a^These models only converged with random intercepts only (no random slopes).

#### Latency Until the Next Mention

The Reconnection with Specific Individuals Hypothesis predicts a *positive* association of relative post popularity and latency until the next mention. However, we found a significant *negative* relationship between the popularity of the last post and the latency until the next mention (Table 1). Compared with the upper two thirds, the slope was less steep in the lowest third (Table 1). This suggests that the negative relationship between the popularity of the previous post and the latency until the next mention is attributable to particularly quick mentions following *more* popular posts (Figure 1).

Within-study replication also shows a significant negative relationship between post popularity and latency until the next mention, which was also reflected in the upper two thirds of popular posts. However, when considering only the lower third of popularity, the relationship between post popularity and mentioning latency was not significantly different from zero (Table 1). The discretizing factor significantly improved model fit, χ(1)^2^ = 18.95, *p* < .001. Our findings suggest a nonlinear relationship between the popularity of a post and the latency until the next mention, with the strongest relationship in the upper two thirds of the distribution. Findings are thus in disagreement with the Reconnection with Specific Individuals Hypothesis.

#### Latency Until the Next Retweet

We found a significant relationship between the popularity of the last post and latency until the next retweet (Table 1). The slope was markedly steeper in the lowest third than the slope in the upper two thirds, for which there was no significant relationship between popularity and latency until the next retweet (Table 1). Within-study replication also showed consistent patterns (Table 2). Introducing the discretizing factor improved model fit, χ(1)^2^ = 8.09, *p* = .004. Taken together, there was a weak but general trend that users retweeted more quickly after less popular posts, in line with the Reconnection with Specific Individuals Hypothesis.

#### Exploratory Analyses Including Extremely Popular Posts

In exploratory fashion, we repeated all analyses with the full data set, that is, not excluding extremely popular posts to gauge generalizability beyond examining unpopular posts. The results were generally highly robust and not affected by including highly popular posts (Table 1).

#### Exploratory Within-Study Replication Analyses

**Figure 2.**
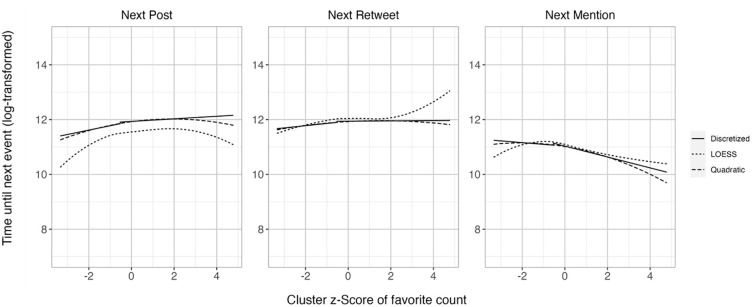
Association of Relative Favorite Count of a Post With the Time Taken Until the Next Own Tweet (Left Plot), Mention (Plot in the Middle), and Retweet (Right Plot), in Study 1, Comparing Discretized (Tertile Split), Quadratic Model, and LOESS Curves.

To replicate the results, we used a random subsampling method (Molinaro et al., 2005), drawing five random samples of *N* = 1,000 from the total sample to assess the results’ consistency across subsamples. The vast majority of relationships were robust (Table 2). Given that the quadratic continuous predictor proved to be a good fit (see OSF), Figure 2 shows the discretized tertile split, a model with continuous quadratic predictor, and the LOESS (locally estimated scatterplot smoothing) curve, a regression curve that is estimated within smaller subsets and can thus display nonlinear functions without a priori assumptions (Cleveland, 1979). The quadratic term was significant for almost all analyses (Table 1), indicating nonlinear relationships between the favorite count and the latency until the next post, the next mention (only in the filtered data set), and the next retweet. All quadratic coefficients are negative. For latency until the next post and for latency until the next retweet, this means that both after very unpopular posts and after very popular posts, users post and retweet again more quickly. For the latency until the next mention, the negative quadratic coefficient indicates that both after very unpopular posts and after very popular posts, users mention others again more quickly.

#### Between-Person Effects of Follower Count and Tweet Rate

As preregistered, we also tested whether the average tweet rate of the user (i.e., how often a user posted per day, only considering own content) and/or the number of followers of the user moderates the tested relationships. Analyses of tweet rate indicated that for the latency until the next mention, the effect of favorites was stronger for users with a lower tweet rate (Table 3). Analyses of follower count indicated that for the latency until the next mention and until the next retweet (only filtered data set), the effect of favorites was stronger for users with fewer followers (Table 3).

**Table 3.** Between-Person Effects Including Tweet Rate and Follower Count, in Study 1.

Sample	Latency variable	*Favorites* [95% CI]	*Favorites*—% (+1 SD/−1 SD)	*Tweet rate* [95% CI]	*Tweet rate*—% (+/−1 tweet/day)	*Favorites × Tweet rate* [95% CI]	*Favorites × tweet rate*—% (+/−1 SD *×* 1 tweet/day)	Random effects (*SE*)
Filtered	Next post	**0.10 [0.08, 0.12]**, ***p* < .001**	**+10.73%/**−**9.70%**	−**0.65 [**−**0.69**, −**0.62]**, *p* **< .001**	**+92.32%/**−**48.00%**	0.00 [−0.01, 0.01], *p* = .577	+92.32%/−48.00%	*u_0j_* = 1.56; *r_ij_* = 3.51
	Next mention	−**0.16 [**−**0.18**, −**0.14]**, *p* **< .001**^†^	−**14.79%/+17.35%**	−**0.51 [**−**0.56**, −**0.46]**, *p* **< .001**	−**40.01%/+66.70%**	**0.02 [0.00, 0.03]**, *p* **= .001**	**+2.02%/**−**1.98%**	*u_0j_* = 2.90; *r_ij_* = 5.17
	Next retweet	**0.03 [0.01, 0.05]**, *p* **= .001**	**+3.36%/**−**3.25%**	−**0.49 [**−**0.53**, −**0.45]**, *p* **< .001**	−**38.74%/+63.23%**	0.00 [−0.01, 0.01], *p* = .515	+0.30%/−0.30%	*u_0j_* = 2.34; *r_ij_* = 3.45
Unfiltered	Next post	**0.08 [0.062, 0.102]**, *p* ***<* .001**	**+8.55%/**−**7.87%**	−**0.60 [**−**0.61**, −**0.58]**, *p* **< .001**	−**45.01%/+81.85%**	0.00 [−0.00, 0.02], *p* = .081	+0.80%/−0.80%	*u_0j_* = 0.83; *r_ij_* = 4.16
	Next mention	−**0.23 [**−**0.25**, −**0.20]**, *p* **< .001**^†^	−**20.23%/+25.36%**	−**0.47 [**−**0.48**, −**0.45]**, *p* ***<* .001**^†^	−**37.19%/+59.20%**	**0.03 [0.02, 0.04]**, *p* ***<* .001**	**+2.74%/**−**2.66%**	*u_0j_* = 1.46; *r_ij_* = 6.56
	Next retweet	0.011 [−0.01, 0.033], *p* = .300	+1.11% /−1.09%	−**0.49 [**−**0.51**, −**0.48]**, *p* **< .001**	−**38.92%/63.72%**	0.01 [−0.00, 0.02], *p* = .276	+0.50%/−0.50%	*u_0j_* = 1.38; *r_ij_* = 4.39
Sample	Latency variable	*Favorites* [95% CI]	*Favorites*—% (+1 SD/−1 SD)	*Follower count* [95% CI]	*Follower count*—% (doubling/halving of followers)	*Favorites: follower count* [95% CI]	*Favorites: follower count*—% (+/−1 SD *×* 1 doubling/halving)	Random effects (*SE*)
Filtered	Next post	0.02 [−0.07, 0.11], *p =* .676	+2.02%/−1.98%	−**0.25 [**−**0.28**, −**0.22]**, *p* **< .001**	−**21.81%/+27.89%**	0.01 [−0.00, 0.01], *p* = .072	+0.70%/−0.70%	*u_0j_* = 2.39; *r_ij_* = 3.50
	Next mention	−**0.42 [**−**0.54**, −**0.31]**, *p* **< .001**^†^	−**34.36%/+52.35%**	−**0.19 [**−**0.23**, −**0.16]**, *p* **< .001**	−**17.55%/+21.29%**	**0.02 [0.01, 0.03]**, *p* **< .001**	**+2.22%/**−**2.18%**	*u_0j_* = 3.38; *r_ij_* = 5.18
	Next retweet	−0.07 [−0.17, 0.02], *p =* .141	−7.04%/+7.57%	−**0.19 [**−**0.22**, −**0.15]**, *p* **< .001**	−**17.06%/+20.56%**	**0.01 [0.00, 0.02]**, *p* **= .033**	**+0.80%/**−**0.80%**	*u_0j_* = 2.78; *r_ij_* = 3.45
Unfiltered	Next post	0.01 [−0.09, 0.11], *p =* .78	+1.41%/−1.39%	−**0.21 [**−**0.22**, −**0.20]**, *p* **< .001**	−**18.78%/+23.12%**	0.01 [−0.00, 0.01], *p* = .129	+0.60%/−0.60%	*u_0j_* = 1.39; *r_ij_* = 4.53
	Next mention	−**0.44 [**−**0.57**, −**0.32]**, *p* **< .001**^†^	−**35.79%/+55.74%**	−**0.15 [**−**0.16**, −**0.14]**, *p* ***<* .001**^†^	−**13.84%/+16.07%**	**0.02 [0.01, 0.03]**, *p* ***<* .001**	**+1.92%/**−**1.88%**	*u_0j_* = 1.75; *r_ij_* = 6.80
	Next retweet	−0.05 [−0.16, 0.05], *p* = .334	−5.07%/+5.34%	−**0.17 [**−**0.18**, −**0.16]**, *p* **< .001**	−**15.63%/+18.53%**	0.01 [−0.00, 0.01], *p* = .197	+0.50%/−0.50%	*u_0j_* = 1.62; *r_ij_* = 4.65

*Note.* Significant effects are bold-faced. ^†^ Random-intercept only model. The *Follower Count* was transformed with a logarithm of base 2, for simple interpretation of the effect size, as 1 unit equals a doubling or halving of the *Follower Count.*

### Discussion

In Study 1, we investigated user behavior on Twitter, examining the relationship between the popularity of posts and time until the next user activity. One striking observation is that users tend to post more quickly following an unpopular tweet, potentially as a way to regain lost connection in line with the Reconnection with Many Hypothesis and contradicting the Withdrawal Hypothesis. As an alternative interpretation of the observed effects, users may post again more quickly in an attempt to influence the Twitter algorithm (i.e., if one post receives many favorites, another post is also more likely to be displayed in other users’ feeds because it is similar content). Put differently, posting again quickly may be a strategic way to increase exposure to the initial Tweet. We address this limitation with Study 2 because on Reddit, posts are displayed chronologically in specific subreddits, and therefore, posting again quickly does not influence the algorithm in the same way as it would on Twitter.

We observed a weaker, but comparable trend in the latency until the next retweet: Users retweet more quickly the less popular their previous post was, in line with the Reconnection with Specific Individuals Hypothesis. However, the strength of this relationship was not clearly intensified in the lower third of the distribution, suggesting that retweet latency is either less impacted by having an unpopular tweet, or more impacted by having a popular tweet.

Interestingly, when it came to the latency of mentions, the pattern reversed: There was a negative relationship between the popularity of a previous post and the latency until the next mention, meaning that individuals mentioned others more quickly after more *popular* posts. This trend was weaker in the lowest third of the popularity distribution, which differed from the patterns observed for posting and retweeting. Thus, the observed mentioning pattern does not align with the Reconnection with Specific Individuals Hypothesis. However, we caution against drawing definitive conclusions from this finding because the positive relationship could be a result of decreased time until the next activity in response to one’s own more popular post. That is, after having a particularly popular post, many others may comment on that post, resulting in more mentions when the users interact with those comments (e.g., “*@*user, thank you for your comment!”). Therefore, quicker mentioning activity after a popular post does not necessarily reflect an active engagement with other users; instead, the engagement could be reactive, in line with reciprocity norms (Gouldner, 1960). We aim to delineate this finding further in Study 2 by focusing on commenting latency that is unrelated to one's own post.

We also tested for quadratic relationships between favorite count and latency until the next behavior, finding support for quadratic relationships for all latency variables. This indicates that users post, retweet, and mention others more quickly after very unpopular and after very popular posts. This observation aligns with both our Reconnection Hypotheses and with previous findings that individuals use a reward-learning approach to using social media: Getting many favorites is rewarding and may prompt individuals to use social media more to obtain more psychological reward (Lindström et al., 2021). Importantly, the results were largely consistent when extremely popular posts were not filtered out, speaking in favor of the generalizability of the results.

Moreover, we found that users who posted less frequently and had fewer followers reacted more strongly to having an unpopular tweet in that they posted, mentioned, and retweeted others more quickly. Put differently, those who post more frequently and those who have more followers seem to be less affected by having one unpopular post. This could mean that the emotional impact of a single post’s performance is lower for frequent and for posters with a big followership (e.g., E. Lee, Lee, et al., 2015). Moreover, if follower count can be interpreted as an indicator of social power on Twitter, our findings align with previously found buffering effects of higher social power against the negative effects of social exclusion (Kuehn et al., 2015). However, the between-person effects of tweet rate and follower count were not consistent across the dependent variables and, therefore, have to be interpreted cautiously.

In summary, rather than observing a general increase or decrease in time until the next activity following an unpopular post, we found that the time until more public activities (posting and retweeting, which reach all of one’s followers) tended to increase, while the time until activities aimed at specific others (mentions, which directly address only one user) tended to decrease. This might indicate that users are looking to rebound from the lack of crowd affirmation (reflected in the number of favorites) following an unpopular post, in line with the Reconnection with Many Hypothesis, as opposed to seeking validation from individual users, in what would be in line with the Reconnection with Specific Individuals Hypothesis.

## Study 2

Study 2 extends Study 1 in multiple ways by examining another context, Reddit. Reddit users may post links to articles, videos, or other content, and other users can vote on the content’s popularity. Reddit is organized in *subreddits* that are focused on specific topics. This offers the chance to look at commenting latency in *other* subreddits, that is, posting comments that are not directly related to one’s own content and addressing a major limitation of Study 1. Another distinction is Reddit’s emphasis on user anonymity. While both Twitter and Reddit can be used anonymously in theory, many users choose a Twitter profile name that makes them identifiable to some extent (e.g., Peddinti et al., 2014). Reddit is one of the most anonymous platforms at the time of this contribution (e.g., Triggs et al., 2021). Anonymity may play a key role in how individuals use social media: Users with a higher degree of anonymity post more (e.g., Peddinti et al., 2014) and more honest content (e.g., Karahanna et al., 2018). Thus, identifying whether behavior following unpopular posts differs based on users’ anonymity was another important endeavor of Study 2.

Users can upvote or downvote content on Reddit. To achieve comparability with the relative favorite count on Twitter used in Study 1, we used the sum of upvotes and downvotes (i.e., *score*) that is displayed in Reddit as the predictor variable in this study.

We test the following preregistered hypotheses in Study 2:

**Hypothesis 1:** “Reconnection with Many”: Submissions with a lower (vs higher) within-person-centered score have smaller latency until the next submission (a) in any subreddit and (b) in the same subreddit (i.e., the one of the previous submission).
**Hypothesis 2**: “Reconnection with Specific Individuals”: Submissions with a lower (vs higher) within-person-centered score have smaller latency until the next comment in another subreddit.^3^

Again, we also preregistered the assumption that the effects described in Hypotheses 1 and 2 will be significantly stronger for submissions at the lower end of the distribution of scores, indicating that effects are driven by unpopular rather than average or very popular submissions. For Study 2, we explicitly preregistered splitting the distribution into the lower third and the two upper thirds of the distribution.

Given the evidence against the Withdrawal Hypothesis (Hypothesis 3) in Study 1, we did not preregister the Withdrawal Hypothesis in Study 2.

### Method

#### Social Media Data

For Study 2, we obtained data from 2,000 Reddit users in 10 different subreddits (i.e., thematic collections of posts). We chose the 10 subreddits with the most followers at the time of preregistration (i.e., March 2023) that had at least 200 users who contributed 10 or more posts (i.e., “funny,” “aww,” “todayilearned,” “pics,” “Showerthoughts,” “food,” “EarthPorn,” “Art,” “gifs,” and “OldSchoolCool”) that mainly contained individual content creator’s own content (e.g., no help requests or sharing of official content—for a more detailed list, see the subreddit selection script via OSF Supplement 2c). For each of the subreddits, we sampled frequent contributors to this subreddit (≥10 contributions). For each user, we exclusively analyzed posts from the subreddit where they were frequently active. As our dependent variables, we used latencies until users’ next post, considering posts within the same subreddit and those in other subreddits, and we used latency until the next comment in another subreddit. The final data set consisted of 58,442 posts from 2,000 users and posted between November 2008 and May 2023.

#### Measures

The measures in Study 2 mirrored the measures in Study 1, with minor differences due to differences between Twitter and Reddit.

##### Score

Different from Twitter, Reddit users can choose to either upvote or downvote a post. However, on Reddit, a post’s visibility is disproportionally influenced by its upvote count (see https://www.reddit.com/r/blog/comments/o5tjcn/evolving_the_best_sort_for_reddits_home_feed/?rdt=50386). Posts with many downvotes become less visible, as the algorithm prioritizes posts with more upvotes. Posts receive downvotes only when they are still visible, that is, when they are popular. Consequently, a high number of downvotes are often an indicator of a post’s reach and visibility. Therefore, we used the sum score of upvotes and downvotes that is also displayed on Reddit. Note that all scores in the data set were positive. The score was transformed in the same fashion as the favorite count in Study 1.

##### Latency Until Next Post

Due to Reddit’s subreddit structure, we can differentiate between users’ behavior in the same subreddit versus in other subreddits. We, therefore, differentiate between latency until the next post *in any subreddit* and *in the same subreddit as the last post*.

##### Latency Until Next Comment (Other Subreddit)

We analyze latency of the next comment *in any other subreddit*, that is, other than the one where the respective post was posted. This allows us to control for shorter latencies of commenting due to the popularity of a previous post, which we could not control for in the Twitter data. Comments in Reddit are similar to mentions in Twitter, as both signify direct responses to another person’s post.

### Results

#### Sample Characteristics

Users received a median score of 56.98 (*M* = 513.77) on their posts in the subreddit they were sampled from and the median user posted 0.98 times a week (*M* = 2.48) in any subreddit. Users posted 15.37% of their posts in the subreddit they were sampled from on average (for further descriptive analyses see OSF Supplement 2a).

#### Latency Until the Next Post

##### In Any Subreddit

**Figure 3.**
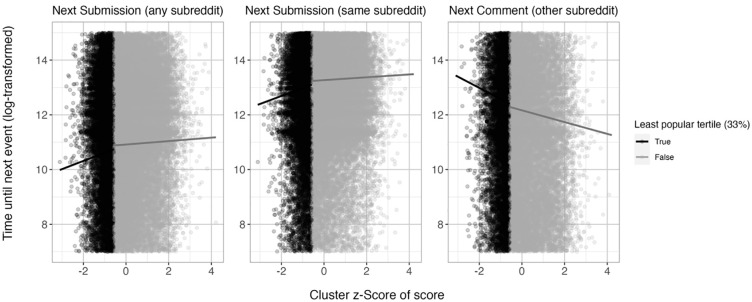
Association of Relative Score of a Post With the Time Taken Until the Next Post in Any Subreddit (Left Plot), the Next Post in the Same Subreddit (Plot in the Middle), and the Next Comment in Another Subreddit Than That of the Previous Post (Right Plot), in Study 2. *Note.* The slopes in the graphs are based on the regression coefficients of the linear mixed models. Separate slopes are calculated for the least popular third of all posts versus the remaining posts.

We found a significant positive relationship between the popularity of the last post and latency until the next post in any subreddit. We again introduced a discretizing factor, dividing the slope estimates into the lowest third of popularity and the upper two thirds representing the more popular posts. The association was significant for both slopes, but we observed a steeper slope in the lowest third (Table 4). The discretizing factor improved model fit, χ(1)^2^ = 50.55, *p* < .001. Our findings suggest a nonlinear relationship between the score of a post and the latency until the next post in any subreddit, with the strongest relationship appearing in the lower third of the distribution (Figure 3). Thus, users tend to post more quickly following an unusually unpopular post, in line with the Reconnection with Many Hypothesis.

**Table 4.** Results of the LMEMs in Study 2, With Filtered (i.e., Excluding Extremely Popular Posts) and Unfiltered (i.e., Full Data Set) Data.

Data	Latency variable	Main effect *b* [95% CI]	Main effect—% (+1 SD/−1 SD)	Lower third *b* [95% CI]	Lower third—% (+/−1 SD)	Upper two thirds *b* [95% CI]	Lower third—% (+/−1 SD)	Quadratic term *b* [95% CI]	Fixed effects (*SE*)	Random effects (*SE*)
Filtered	Next post (any subreddit)	**0.16 [0.13, 0.19]**, ***p*** **< .001**	**+17.00%/**−**14.53%**	**0.30 [0.25, 0.35]**, *p* **< .001**	**+33.94%/**−**25.89%**	**0.06 [0.02, 0.10]**, ***p*** **= .002**	**+6.45%/**−**6.06%**	−**0.08 [**−**0.09**, −**0.06]**, ***p*** **< .001**^†^	***β_0_* = 10.92 (0.05)**, ***p*** **< .001**; ***β1* = 0.05 (0.02)**, ***p*** **= .002**; ***β2* = 0.24 (0.03)**, ***p*** ***<* .001**	*u_0j_* = 3.47; *u_1j_* = 0.11; Cov(*u_0j_, u_1j_*) = 0.05; *r_ij_* = 7.21
	Next post (same subreddit)	**0.15 [0.12, 0.18]**, ***p*** **< .001**	**+15.84%/**−**13.67%**	**0.29 [0.25, 0.33]**, ***p*** **< .001**	**+33.65%/**−**25.17%**	**0.05 [0.02, 0.09]**, ***p*** **= .003**	**+5.32%/**−**5.05%**	−**0.09 [**−**0.10**, −**0.07]**, ***p*** **< .001**	***β_0_* = 13.27 (0.04)**, ***p*** **< .001**; ***β1* = 0.05 (0.03)**, ***p*** **= .004**; ***β2* = 0.24 (0.03)**, ***p*** **< .001**	*u_0j_* = 2.08; *u_1j_* = 0.15; Cov(*u_0j_, u_1j_*) = −0.22; *r_ij_* = 4.60
	Next comment (other subreddit)	−**0.29 [**−**0.33**, −**0.25]**, ***p*** **< .001**	−**25.32%/+33.91%**	−**0.41 [**−**0.47**, −**0.35]**, ***p*** **< .001**	−**33.60%/50.60%**	−**0.22 [**−**0.26**, −**0.17]**, ***p*** **< .001**	−**19.35%/23.99%**	**0.06 [0.03, 0.08]**, ***p*** ***<* .001**^†^	***β_0_* = 12.16 (0.08)**, ***p*** **< .001**; ***β1* =** −**0.22 (0.03)**, ***p*** ***<* .001**; ***β2* =** −**0.19 (0.04)**, ***p*** ***<* .001**	*u_0j_* = 2.85; *u_1j_* = 0.02; Cov(*u_0j_, u_1j_*) = −0.14; *r_ij_* = 3.36
Unfiltered	Next post (any subreddit)	**0.08 [0.06, 0.11]**, ***p*** **< .001**	**+8.65%/**−**7.96%**	**0.32 [0.25, 0.40]**, ***p*** **< .001**	**+38.23%/**−**27.65%**	**0.05 [0.02, 0.07]**, ***p*** **< .001**	**+4.90%/**−**4.67%**	−**0.01 [**−**0.02**, −**0.00]**, ***p*** **= .003**^†^	***β_0_* = 10.90 (0.04)**, ***p*** **< .001**; ***β1* = 0.08 (0.01)**, ***p*** **< .001**; ***β2* = 0.27 (0.04)**, ***p*** ***<* .001**	*u_0j_* = 3.48; *u_1j_* = 0.5; Cov(*u_0j_, u_1j_*) = 0.06; *r_ij_* = 7.01
	Next post (same subreddit)	**0.07 [0.06, 0.09]**, ***p*** **< .001**	**+7,47%/**−**6.95%**	**0.33 [0.27, 0.39]**, ***p*** **< .001**	**+39.39%/**−**28.26%**	**0.03 [0.01, 0.05]**, ***p*** **< .001**	**+3.25%/**−**3.15%**	−**0.02 [**−**0.03**, −**0.01]**, ***p*** **< .001**	***β_0_* = 13.24 (0.03)**, ***p*** **< .001**; ***β1* = 0.07 (0.008)**, ***p*** ***<* .001**; ***β2* = 0.30 (0.03)**, ***p*** **< .001**	*u_0j_* = 2.00; *u_1j_* = 0.01; Cov(*u_0j_, u_1j_*) = −0.32; *r_ij_* = 4.21
	Next comment (other subreddit)	−**0.35 [**−**0.38**, −**0.32]**, ***p*** **< .001**	−**29.46%/+41.76%**	−**1.14 [**−**1.24**, −**1.04]**, ***p*** **< .001**	−**68.04%/212.90%**	−**0.23 [**−**0.27**, −**0.20]**, ***p*** **< .001**	−**20.90%/26.42%**	**0.06 [0.05, 0.07]**, ***p*** ***<* .001**^†^	***β_0_* = 11.80 (0.08)**, ***p*** **< .001**; ***β1* =** −**0.23 (0.02)**, ***p*** ***<* .001**; ***β2* =** −**0.91 (0.05)**, ***p*** ***<* .001**	*u_0j_* = 11.97; *u_1j_* = 0.16; Cov(*u_0j_, u_1j_*) = 0.11; *r_ij_* = 9.12

*Note*. Significant effects are bold-faced. ^†^ Random-intercept only model. Parameters: intercept (*β_0_*), predictor score (*β1*), discretizing factor (*β2*), intercept variance (*u_0j_*), slope variance (*u_1j_*), covariance (*u_0j_, u_1j_*), residual variance (*rij*).

##### In the Same Subreddit

Parallel findings emerged for the latency until the next post in the *same* subreddit. We observed a significant positive relationship between popularity of the last post and the latency until the next subreddit-specific post. The discretizing factor revealed similar patterns: The association was significant for both slopes, but the slope was steeper in the lowest third (Table 4 and Figure 3). The discretizing factor again improved model fit, χ(1)^2^ = 77.44, *p* < .001, mirroring the findings from the post latency in any subreddit, in line with the Reconnection with Many Hypothesis.

#### Latency Until the Next Comment (Other Subreddits)

Regarding latency until the next comment in other subreddits^4^ (i.e., other than that of the previous post), we found a significant *negative* relationship between the popularity of the last post and the latency until the next comment. This suggests that after posting less popular content in a specific subreddit, users tend to engage in other communities less quickly. Again, the association was significant for both slopes, but the slope was steeper in the lowest third (Table 4). The discretizing factor improved model fit, χ(1)^2^ = 23.18, *p* < .001. Our findings suggest a nonlinear relationship between the popularity of a post and the latency until the next comment in a different subreddit, with the strongest relationship appearing in the lower third of the distribution (Figure 3). Thus, users tend to comment more slowly following an unusually unpopular post, in disagreement with the Reconnection with Single Individuals Hypothesis.

#### Exploratory Analyses Including Extremely Popular Posts

Again, we repeated all analyses with the full data set, not excluding extremely popular posts. Again, the pattern of results was highly robust (Table 4).

#### Exploratory Within-Study Replication Analyses

**Figure 4.**
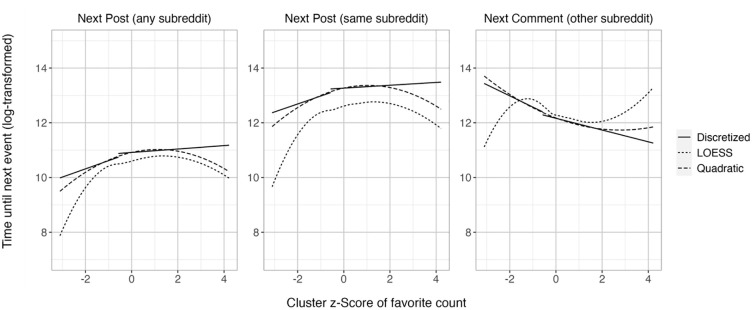
Association of Relative Score of a Submission With the Time Taken Until the Next Post in Any Subreddit (Left Plot), Next Post in the Same Subreddit (Plot in the Middle), and Next Comment in Another Subreddit (Right Plot), in Study 2, Comparing Discretized (Tertile Split), Quadratic Model, and LOESS Curves. *Note.* Even though the LOESS curve for comments may look cubic, LOESS plots that rely on very small samples in the margins are subject to a lot of uncertainty. Indeed, the cubic model is a worse fit than the quadratic and the discretized model, see OSF.

Again, to replicate the results, we used a random subsampling method (Molinaro et al., 2005), finding that the vast majority of relationships were robust (Table 2). Again, given that the quadratic continuous predictor proved to be a good fit (see OSF), Figure 4 shows the discretized tertile split, a model with continuous quadratic predictor, and the LOESS curve. As in Study 1, the quadratic term was significant for all analyses (Table 4), indicating nonlinear relationships between Reddit scores and all latency variables. For the latency until the next submission in any and the same subreddit, the quadratic coefficients were negative, meaning that after very unpopular submissions and after very popular submissions, users post again more quickly in any and the same subreddit. For the latency until the next comment, the quadratic coefficient was *positive*, meaning that after very unpopular submissions and after very popular submissions, users comment in other subreddits more slowly.

#### Between-Person Effects of Submission Rate

As preregistered, we also tested whether the average submission rate (i.e., how often a user posted per day, only considering own content) moderates the tested relationships. Indeed, the effects of score were stronger for users with a lower submission rate for next submission in any subreddit (only filtered data set) and for next comment in another subreddit (Table 5).

**Table 5.** Between-Person Effects Including Submission Rate, in Study 2.

Sample	Latency variable	*Score* [95% CI]	*Score*—% (+1 *SD*/−1 *SD*)	*Submission rate* [95% CI]	*Submission rate*—% (+/−1 submission/day)	*Score × submission rate* [95% CI]	*Score × submission rate*—% (+/−1 *SD ×* 1 submission/day)	Random effects (*SE*)
Filtered	Next submission (any subreddit)	**0.17 [0.14, 0.20]**, ***p*< .001**	**+18.65%/**−**15.72%**	−**0.63 [**−**0.71**, −**0.55]**, ***p*< .001**	−**46.58%/+87.19%**	−**0.04 [**−**0.07**, −**0.01]**, ***p* = .007**	−**3.82%/+3.98%**	*u_0j_* = 3.05; *r_ij_* = 7.21
	Next submission (same subreddit)	**0.15 [0.12, 0.18]**, ***p*< .001**^†^	**+15.72%/**−**13.58%**	−**0.35 [**−**0.41**, −**0.29]**, ***p*< .001**	−**29.60%/+42.05%**	0 [−0.03, 0.03], *p* = 1	0%/0%	*u_0j_* = 1.95; *r_ij_* = 4.61
	Next comment (other subreddit)	−**0.31 [**−**0.35**, −**0.27]**, ***p*< .001**	−**26.51%/+36.07%**	−**0.36 [**−**0.52**, −**0.20]**, ***p*< .001**	−**30.23%/+43.33%**	**0.05 [0.01, 0.08]**, ***p* = .022**	**+4.60%/**−**4.40%**	*u_0j_* = 11.38; *r_ij_* = 8.78
Unfiltered	Next submission (any subreddit)	**0.09 [0.06, 0.11]**, ***p<* .001**	**+9.20%/**−**8.42%**	−**0.64 [**−**0.72**, −**0.56]**, ***p*< .001**	−**47.22%/+89.46%**	−0.01 [−0.04, 0.01], *p* = .234	−1.39%/+1.41%	*u_0j_* = 3.04; *r_ij_* = 7.01
	Next submission (same subreddit)	**0.07 [0.05, 0.09]**, ***p*< .001**^†^	**+7.36%/**−**6.85%**	−**0.35 [**−**0.41**, −**0.29]**, ***p*< .001**^†^	−**29.67%/+42.19%**	−0.00 [−0.02, 0.02], *p* = .894	−0.10%/+0.10%	*u_0j_* = 1.86; *r_ij_* = 4.21
	Next comment (other subreddit)	−**0.37 [**−**0.40**, −**0.34]**, ***p*< .001**	−**30.86%/+44.63%**	−**0.32 [**−**0.48**, −**0.16]**, ***p*< .001**	−**27.60%/+38.13%**	**0.06 [0.02, 0.09]**, ***p* = .001**	**+5.97%/**−**5.64%**	*u_0j_* = 11.73; *r_ij_* = 9.16

*Note.* Significant effects are bold-faced. ^†^ Random-intercept only model.

### Discussion

Study 2 sought to corroborate Study 1’s findings on the relationship between the popularity of posts and time until the next user activity on another platform: Reddit. We found that users tend to post more quickly following an unusually unpopular post, both in any subreddit and in the same subreddit, in line with the Reconnection with Many Hypothesis.

By focusing on comments that are unrelated to one’s own post, Study 2 delineated quicker commenting activity as an artifact of responses to an own popular post. In this study, individuals took a particularly long time before commenting in other subreddits, following highly unpopular posts. Study 2 thus overall aligns with observations from Study 1 in that there is not a general increase or decrease in the time until the next activity following an unpopular post. Rather, activities aimed at reconnecting with many others, such as posting, are performed faster, while activities targeting specific others, such as commenting, are performed more slowly, lending further support to the Reconnection with Many Hypothesis over the Reconnection with Specific Individuals Hypothesis. Again in line with Study 1, all results could be replicated when extremely popular posts were not filtered out. Furthermore, we again found support for quadratic relationships between Reddit scores and all latency variables: After very unpopular submissions and after very popular submissions, users posted again more quickly but commented less quickly. This observation again aligns with the Reconnection with Many Hypothesis and with social media user’s reward-learning approaches after popular posts (Lindström et al., 2021). Moreover, Study 2 also documents that those who post more frequently on Reddit are less affected by having one unpopular post. Interestingly, less frequent posters only posted more quickly in any subreddit, not the same subreddit, which could indicate that they sought the lacking affirmation in a different community.

## General Discussion

Social media and experiences of social exclusion are part of the reality of people’s everyday lives around the globe (e.g., Büttner, Ren, et al., 2025; *Forbes*, 2023). We investigated how users react to social exclusion online, using observational data from two large social media platforms, Twitter and Reddit. Both studies show that having a relatively unpopular post on social media motivates users to post again more quickly to a broad audience, in line with what we term the Reconnection with Many Hypothesis. In Study 1, this means that Twitter users who just experienced having a relatively unpopular tweet (i.e., by one standard deviation, in the lowest third of popularity), post again more quickly by 14.69% of the average time until the next post (Table 1). In Study 2, the effects were even stronger: For instance, a Reddit user who just experienced having a relatively unpopular Reddit post (i.e., by one standard deviation, in the lowest third of popularity) posts again more quickly by 25.89% of the average time until the next submission in any subreddit, and more quickly by 25.17% of the average time until the next submission in the same subreddit (Table 4). However, exclusion does not motivate users to target specific others, such as via comments or mentions.

Our finding that there is no general increase in the time until any next activity following a relatively unpopular post may be interpreted as a form of acknowledgment-seeking that is specific to experiencing exclusion on social media. Rather than a general need to reconnect with others, be that, a broad audience or specific others, exclusion on social media seems to specifically motivate acknowledgment-seeking actions like posting content again quickly. In line with the Reconnection with Many Hypothesis, our findings speak in favor of individuals behaving to be *acknowledged* rather than to be *liked* after social media exclusion. By posting more quickly after an unpopular post, social media users may regain acknowledgment from others (Karahanna et al., 2018) and re-plenish needs that are threatened by exclusion (e.g., Lutz et al., 2022; Williams, 2009). Furthermore, as hypothesized, the effects were stronger in the least popular third of posts, suggesting that the effect was driven by the difference in responses following unpopular versus regular posts, rather than regular versus popular posts.

Reversely, individuals seem to refrain from targeting specific others to reconnect, such as via comments or mentions, lending no support to the Reconnection with Specific Individuals Hypothesis. Potentially, targeting specific others entails a higher social risk of being excluded again because specific individuals might (intentionally or unintentionally) ignore a comment or mention. This aligns with findings that targets of exclusion are hesitant to share their experiences with others because they fear further exclusion (e.g., Meral et al., 2021). Moreover, even though the degree of a user’s anonymity may influence their social media use (e.g., Karahanna et al., 2018; Peddinti et al., 2014), we observed that it does not matter whether the excluded users were completely anonymous (Reddit) or not (Twitter).

We here focus on the *relative* unpopularity of users’ content as an indication of social exclusion, comparing the number of paralinguistic digital affordances (here: favorites on Twitter and upvote-downvote-scores on Reddit, Hayes et al., 2016) that users received to the number of paralinguistic digital affordances they usually received. We, therefore, focused on having a relatively unpopular post, rather than general behavior on social media. Previous experimental research has extensively shown that receiving fewer paralinguistic digital affordances (e.g., Likes) than others or receiving no Likes at all is perceived as exclusionary and threatens psychological needs that are typically threatened after exclusion (e.g., Lutz & Schneider, 2020; Reich et al., 2018). We extend this approach by using the popularity of users’ posts in light of to the *usual* popularity of their posts, that is, *relative* popularity. Relative popularity might be more informative than the absolute number of Likes since individuals who usually receive few Likes might experience psychological benefits even from receiving one or two Likes. This idea aligns with the concept of “Power of One” holding that being included even by one person alleviates the sting of exclusion (e.g., Büttner, Jauch, et al., 2024; DeWall et al., 2010).

Although not the focus of this contribution, we also find that users with very popular posts engage more quickly in acknowledgment-seeking behaviors. This aligns with recent work by Lindström et al. (2021) showing that individuals approach social media with a reward-learning mindset so that more popular posts lead to faster posting.

The present findings rely on individuals who post social media content with a minimum frequency. As such, these findings may generalize better to individuals who post regularly as opposed to those who post rather infrequently. Still, we believe that our findings generalize to any social media user who regularly posts content. We focused on two mainly text-based platforms, Twitter and Reddit; however, image-based platforms such as Instagram or Facebook may carry more emotionality and may be more intimate than text (e.g., E. Lee, Lee, et al., 2015; Pittman & Reich, 2016). Social media research has to be mindful of the ephemerality of single platforms and their features when making statements about generalizability (see Büttner et al., 2023 for a discussion). We believe that our findings generalize to other, also future, social media platforms, as long as they entail user-generated posted content and have mechanisms of paralinguistic affordance that signal approval (e.g., Hayes et al., 2016).

The present findings are silent about the effectiveness of different behaviors following exclusion on social media. The effectiveness of posting again quickly after exclusion on social media for recovering threatened needs may depend on multiple factors, such as who reacts to the post, how quickly other social media users react, but also, trait-level moderators of the excluded user, such as mental health factors (e.g., Büttner et al., 2021), or chronic experiences of exclusion (Büttner, Jauch, et al., 2024, Study 2). Future research could combine individuals’ social media data (e.g., Zhao et al., 2023) with experiments (e.g., Lindström et al., 2021) to examine users’ reactions to exclusion and effects of social media activity on recovery.

Another avenue for future research is exploring the content of unpopular posts. The present findings are silent about the degree of psychological threat that each exclusion on social media conveys. How hurtful exclusion is and how individuals behave afterward might, at least partly, depend on the emotionality of the unpopular post and the level of need threat elicited by the exclusion experience (Büttner, Ren, et al., 2025). Just as being briefly ignored in an elevator may be easier to recover from than being ignored by a loved one for extended periods of time (e.g., Williams, 2009), having an emotionally important social media post ignored might hurt more and have other effects on behavior. Relatedly, future research should examine under which circumstances individuals turn to social media following exclusion experiences in real life.

Future research may use text analyses to probe for the degree of pro- or antisociality of posts and comments following social exclusion. Exclusion may drive antisocial behavior such as aggression or violence, mainly because antisocial behavior alleviates threats to power and provocation needs by provoking acknowledgment (e.g., Ren et al., 2018). If social media exclusion indeed mainly triggers acknowledgment-seeking behavior, then social media exclusion may have important implications for antisocial behavior such as trolling, hate speech, or cyberbullying (e.g., Castaño-Pulgarín et al., 2021; Yokotani & Takano, 2021).

Feeling excluded on social media and posting again more quickly could lead to a downward spiral of not getting the attention that one craves and posting again more quickly to be acknowledged by others. Specifically, if a user’s post received comparatively little attention and they post faster as a way of coping, that post could receive comparatively little attention as well because the post is qualitatively inferior, or, because other users may feel bothered by someone who posts a lot of content quickly (i.e., spammers, e.g., Kang et al., 2013). This spiral may foster social media addiction over time (e.g., Poon, 2018).

## Conclusion

Experiences of social media exclusion decrease the time until users post again, likely as a form of acknowledgment-seeking. Conversely, the time until efforts are taken to reconnect with specific others increases. Using large-scale social media data from two different platforms, we provide a broad perspective on online exclusion’s behavioral consequences, contributing to our understanding psychological implications of online communication dynamics.

## Notes

1. Robustness tests (see OSF Supplement 2a, l. 618ff and Supplementary robustness tests table) indicate that the pattern of results in Study 2 remains the same when mimicking exclusion of extremely popular posts in Study 1 and excluding only the 13% most popular Reddit posts.

2. At the time of data collection, user verification (blue ticks) was not available for purchase yet.

3. We also preregistered additional analyses of the count of comments in the 24 hr and the potential moderating effect of submission rate. These analyses are reported as supplementary: OSF.

4. We preregistered to analyze latency until the next comment in any subreddit; however, controlling for posts in the same subreddit allows us to ensure that decreased latencies do not originate from the popularity of a post itself (e.g., engaging with others under one’s own popular post). Analyses with any subreddit and same subreddit can be found online (OSF).
